# Formation of linearly linked Fe clusters on Si(111)-7 × 7-C_2_H_5_OH surface

**DOI:** 10.1186/1556-276X-9-377

**Published:** 2014-08-03

**Authors:** Wanyu Ding, Dongying Ju, Yuanyuan Guo, Ken-ichi Tanaka, Fumio Komori

**Affiliations:** 1 Advance Science Center of Saitama Institute of Technology, Fukaya 369-0293, Japan; 2 School of Materials Science and Engineering, Dalian Jiaotong University, Dalian 116028, China; 3 School of Material Science and Engineering, University of Science and Technology Liaoning, Anshan 114051, China; 4 Institute for Solid State Physics, University of Tokyo, Tokyo, Chiba 277-8581, Japan

**Keywords:** STM, Fe clusters, Single magnetic domain

## Abstract

The Fe atoms were deposited on the Si(111)-7 × 7 surface, which has been saturated with the C_2_H_5_OH molecules. Then, the Fe clusters were formed on Si(111)-7 × 7-C_2_H_5_OH surface and *in situ* observed by the scanning tunneling microscopy (STM). The STM images showed that with the increase of Fe clusters, the size of clusters was about 5 nm and they self-assembled in straightly linked chain crossing the step to lower or upper terrace. X-ray photoelectron spectroscopy (XPS) was *in situ* carried out on the surface of Fe/Si(111)-7 × 7-C_2_H_5_OH samples before and after the introduction of thin air (4.5 × 10^-2^ Langmuir) into the STM chamber. The XPS results showed that the Fe clusters are stable in the abovementioned thin air condition at room temperature. Based on the STM and XPS results, the driving force making one-dimensional straightly linked chain structure might be the magnetic force of the Fe clusters. The formation of straightly linked Fe clusters chains suggests the formation of single magnetic domain Fe clusters.

**PACS:**

07.79.Cz, 81.15.-z, 75.75.Fk

## Background

Based on the phenomenological theory of ferromagnetic material, the conception of magnetic domain was first proposed by P. E. Weiss in 1907 [1], and the structure of magnetic domain based on the interaction of the magneto-static energy was proposed by L. D. Landau and E. M. Lifshitz in 1935 [2]. Recently, it was found that the particles change to single-domain magnetic clusters by decreasing their size [3-5]. Accordingly, the preparation of single magnetic domain clusters is an interesting challenge to magnet materials for high-density magnetic recording medium. So far, the reported critical sizes for single magnetic domains were 85 nm for Ni, 40 nm for Fe_3_O_4_, and 16 nm for α-Fe [3-5], and the cluster with a size lower than the critical value displays super paramagnetism, which could not be applied for the magnetic recording medium. To improve the density of magnetic recording without the restriction of super paramagnetism, it is necessary to prepare the single magnetic domain clusters with limited critical size. From this view point, the Fe single magnetic domain clusters have become the research focus, which could be analyzed for the spin in physics, controllable surface reaction in chemistry, for example, FeN and FeO_*x*_ with the critical size lower than 10 nm.

The Fe clusters were prepared by many techniques, such as chemical precipitation, thermal decomposition, hydrothermal method, sol–gel, and so on [6-9]. The uniformity of cluster size and agglomeration of clusters are difficult to control in these preparation techniques. Therefore, the controlled preparation with uniform size is desired not only for the fundamental studies but also for the application of high-density magnetic recording medium. We intended to prepare the Fe clusters with single magnetic domain by depositing the Fe atoms on Si(111)-7 × 7 surface saturated with ethanol (C_2_H_5_OH). A unit cell of Si(111)-7 × 7 surface is composed of triangular-shaped faulted and unfaulted half unit cells. The half unit cell has six Si ad-atoms and three Si-rest atoms. When the clean Si(111)-7 × 7 surface is exposed to C_2_H_5_OH, C_2_H_5_OH molecules dissociate at the Si ad-atom/Si-rest atom pair sites with almost perfect accuracy, where the Si ad-atom changes to the Si-OC_2_H_5_, the Si-rest atom changes to Si-H, and the saturated Si(111)-7 × 7-C_2_H_5_OH was formed. The formation of Fe clusters on Si(111)-7 × 7-C_2_H_5_OH surface is controlled by the uniformly distributed Si ad-atoms in half unit cells, and we expect the formation of single magnetic domain Fe clusters. In the present work, the Fe atoms were deposited on the surface of Si(111)-7 × 7-C_2_H_5_OH at room temperature, then the growth and distribution of Fe clusters were systematically studied.

## Methods

In our experiments, the Fe clusters were deposited and observed by JSPM-4500S ultra-high vacuum scanning tunneling microscopy (STM) system (JEOL Ltd., Akishima-shi, Japan). The single-crystal n-type Si(111) substrates were firstly ultrasonically pre-cleaned in acetone, ethanol, and deionized water, respectively, and then dried with N_2_ gas. Finally, the substrates were loaded onto the sample holder and placed into the exchange chamber of STM system. After the base vacuum of exchange chamber was less than 5.0 × 10^-4^ Pa, the sample holder was transferred into the treatment chamber. After the baking and degas process for 24 h, the sample holder was translated into the main chamber for STM observation, where the vacuum was about 1.0 × 10^-8^ Pa. Then, the Si(111)-7 × 7-reconstructed surface was obtained according to the standard heating and flashing procedures [10-12]. In order to avoid the chemical reaction of deposited Fe with Si substrate, the substrate surface was passivated by the adsorption of C_2_H_5_OH in the main chamber according to the reported procedures [13]. After the preparation of Si(111)-7 × 7-C_2_H_5_OH surface, it was translated into the treatment chamber, and the Fe atoms were evaporated by heating a W filament with Fe wire. The different amount of Fe atoms was deposited by controlling the deposition time. After the deposition of Fe atoms, the Fe/Si(111)-7 × 7-C_2_H_5_OH sample was translated into the main chamber for STM observation. In order to know the chemical stability of the sample, the sample was exposed to the thin-air condition with 4.5 × 10^-2^ Langmuir (~10^-2^ L for O_2_) in the main chamber by the needle valve. Before and after the exposing, the Fe/Si(111)-7 × 7-C_2_H_5_OH sample was translated into the composition test chamber, respectively, where the sample was *in situ* tested by the GammadataScienta SES-100 X-ray photoelectron spectroscopy (XPS) system (Pleasanton, CA, USA). In our experiments, the XPS spectra were *in situ* performed with an Al_*k*α_ line source (*hv* = 1,486.6 eV) at an incident angle of 45°. Before the measurement, the XPS system was calibrated by the standard Au and Cu samples. In consideration of the signal-to-noise ratio of data, the area of XPS measurement was kept as 100 μm in diameter for all tests. Then, the high-resolution spectra were recorded with 29.35 and 0.125 eV in the pass energy and step, respectively. All spectra were referenced to C *1 s* peak of 284.6 eV.

## Results and discussion

**Figure 1:**
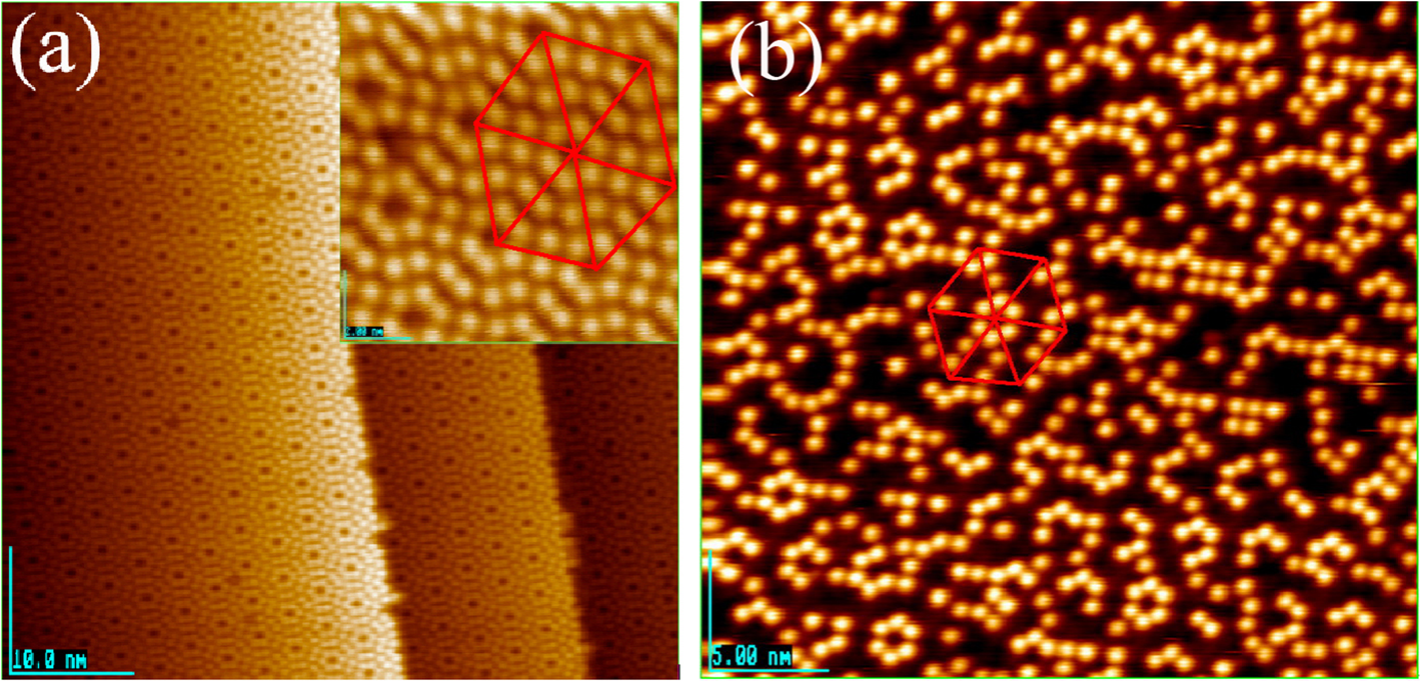
**Typical and standard STM image of Si(111)-7 × 7-reconstructed surface.** The typical STM image of Si(111)-7 × 7-reconstructed surface **(a)**, where the inset was the high magnification. And the standard Si(111)-7 × 7-reconstructed surface saturated by C_2_H_5_OH **(b)**. During all scanning process, the bias voltage and tunneling current was kept at 1.5 V and 0.19 nA, respectively.

Figure 1a shows the typical STM image of Si(111)-7 × 7-reconstructed surface with 55 × 55 nm^2^, where the inset was the high magnification with 10 × 10 nm^2^. In the inset of Figure 1a, each triangular half unit cell contains six Si ad-atoms, which are shown as the bright dots. Figure 1b shows the standard Si(111)-7 × 7-C_2_H_5_OH surface with 25 × 25 nm^2^ and 0.5 mono layer (ML). In Figure 1b, each triangular half unit cell contains three Si ad-atoms and three Si-OC_2_H_5_, which the Si ad-atoms show as the bright dots and Si-OC_2_H_5_ is not shown in the STM image. From Figure 1, it can be confirmed that the Si(111)-7 × 7 and Si(111)-7 × 7-C_2_H_5_OH surface has been prepared by our standard heating, flashing, and saturating procedures [10-13].

**Figure 2:**
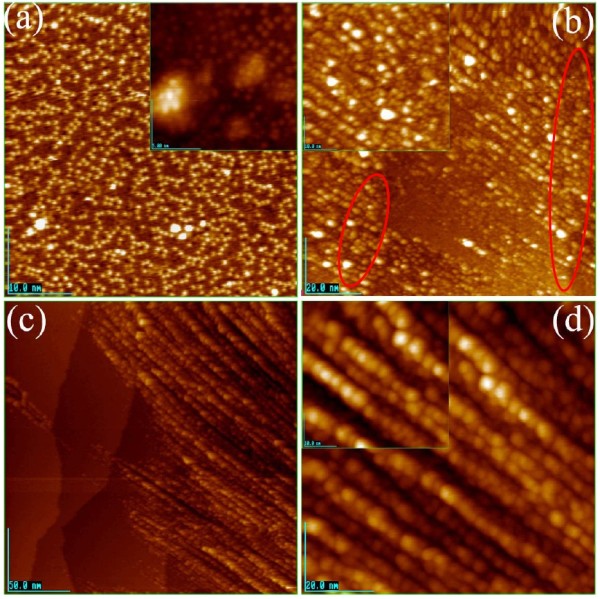
**The STM images of Fe on Si(111)-7 × 7-C**_**2**_**H**_**5**_**OH, which was deposited with a different time. (a)** 0.01 ML, where the inset was the high magnification with 15 × 15 nm^2^. **(b)** 2 ML, where the inset was the high magnification with 35 × 35 nm^2^. **(c) (d)** 4 ML, where the inset was the high magnification with 35 × 35 nm^2^.

The STM images of Fe clusters formed on Si(111)-7 × 7-C_2_H_5_OH surface are shown in Figure 2. From Figure 2a, it can be seen that with 0.01 ML Fe atom deposition, a few of Fe clusters are randomly formed on the Si(111)-7 × 7-C_2_H_5_OH surface, instead of dispersed single Fe atoms. From the inset of Figure 2a, it can be recognized that a Fe cluster having six Fe atoms is formed and the cluster looks to take a pentagonal base pyramid structure [14,15]. When the Fe atom deposition was increased to 2 ML, some Fe clusters linked in straight chains as shown in Figure 2b. It is worthy of note that the straightly linked chain looks to grow to the lower or upper terrace on the Si(111)-7 × 7-C_2_H_5_OH surface by crossing the step edges as indicated in the red circle in Figure 2b. When the Fe deposition was increased to 4 ML, almost Fe clusters were self-assembled by forming straightly linked chain structures as shown in Figure 2c,d.

**Figure 3:**
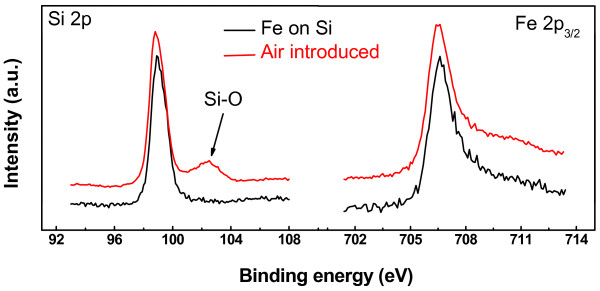
**High-resolution XPS spectra of Si *2p *and Fe *2p***_***3/2 ***_**before and after introduction of air (N**_**2**_ **+ O**_**2**_**) (2 ML).**

Another focus of our report is the chemical stability of Fe clusters in the abovementioned thin-air condition, which are formed on the Si(111)-7 × 7-C_2_H_5_OH surface with straightly linked chain structures. Only with the satisfactory chemical stability in the abovementioned thin-air condition, the Fe clusters with straight chain structures could be applied for the magnetic recording medium. So, the XPS measurement was carried out. Figure 3 shows the high-resolution XPS spectra of 2 ML Fe atom deposited sample and after exposing to O_2_ for ~10^-2^ L in the main chamber. The peaks of Si *2p* and Fe *2p*_*3/2*_ appeared at 98.9 and 706.5 eV, which belong to the Si-Si and Fe-Fe bonds, respectively [16], and the full width at half-maximum (FWHM) value for Si *2p* and Fe *2p*_*3/2*_ spectra implies the single chemical state of Si and Fe atoms. Both the peak position and the FWHM value indicate that the Fe and Si keep the original state, which means no reaction of Fe atoms with Si ad-atoms occurs. By exposing the sample to O_2_ for ~10^-2^ L in the main chamber, no change of the Fe *2p*_*3/2*_ spectrum was observed, whereas a weak shoulder peak appears on the Si *2p* spectrum at about 102.5 eV, which belongs to Si-O bond [16]. Based on the XPS results, one conclusion could be deduced that the Fe clusters are stable in the abovementioned thin-air condition at room temperature. Then, by the precise control of species and concentrations in the main chamber, the chemical reaction on surface of Fe clusters are mild and controllable, which is hopefully to synthesize the FeN_*x*_ and FeO_*x*_ particles with the critical size lower than 10 nm in the future.

From the STM and XPS results, one interesting question is the driving force making linked Fe clusters in a straight chain structure. Attractive force forming Fe clusters and the force making straight chain should be different. Based on the theory of total cohesive energy of cluster in the free space, the lowest energy structures for transition metal cluster was not the layer structure, but the polyhedron structure [14,15]. In fact, the Fe particles prepared at room temperature in the free space have the body-centered cubic structure (<912°C). As the (100) face had the lowest surface energy, the Fe clusters are surround by the (100) face [17]. In this case, the shape of Fe clusters is controlled by the thermodynamic stability of the planes in growth. But if the growth is controlled by the growing rate of crystal planes, the morphology of particles are changed depending on the condition [18].

**Figure 4:**
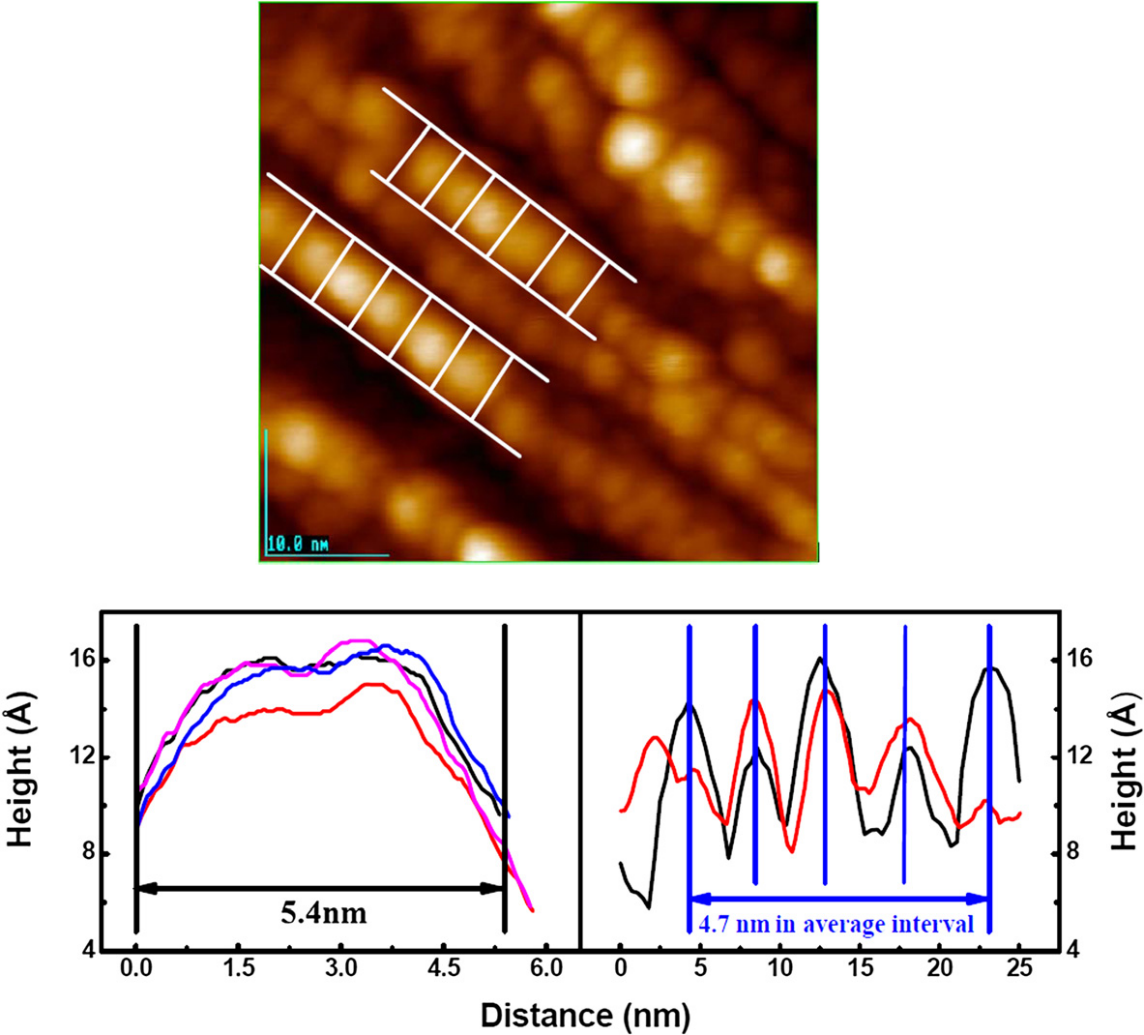
The size of Fe clusters with 4 ML.

As mentioned above, three Si ad-atoms are remained in each half unit cell on the Si(111)-7 × 7-C_2_H_5_OH surface, and the deposited Fe atom may be stabilized by the dangling bond with one electron. In fact, the single-Fe atoms are recognized on the surface at low Fe coverage as shown in Figure 2a. If the Fe atoms are increased in a half unit cell, some kinds of interaction between the Fe atoms stabilize a cluster such as a pentagonal-base pyramid structure observed in the insert of Figure 2a. It should be reminded that the internal bond of Fe clusters may be stronger than the interaction of Fe cluster with the surface, so that small Fe clusters grow instead of the Fe layers [19]. Figure 4 shows the simplified periodic grid of clusters in Figure 2d and the sizes of clusters. From Figure 4, it is known that the linearly arrayed Fe clusters take a size of about 5.4 × 4.7 nm, which is much smaller than the reported critical size of Fe single magnetic domain clusters (~10^1^ nm) prepared by the chemical methods. The 5-nm Fe clusters formed on Si(111)-7 × 7-C_2_H_5_OH showed unusual one-dimensional self-assembly with a regular periodic arrangement as shown in Figure 2c,d, which indicate some kinds of attractive interaction of large Fe clusters along the strings. This fact suggests the possibility for the preparation of ca. 5-nm-size single magnetic domain Fe clusters. It is worthy of note that the straightly linked chain structures appears on larger Fe clusters, just as shown in Figure 2c,d, and the authors presumed the formation of single magnetic domain of 5-nm Fe clusters. In addition, if we could oxidize and/or azotize the 5-nm Fe cluster, we could prepare the strong magnetic materials of FeO_*x*_ and/or FeN_*x*_ with single magnetic domain.

**Figure 5:**
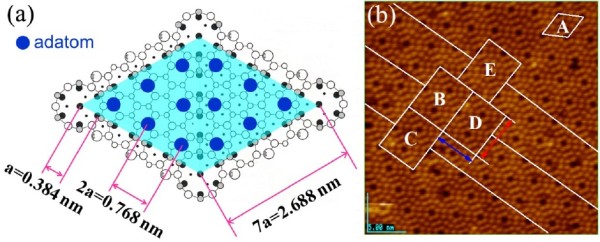
**DAS model of Si(111)-7 × 7-reconstructed surface and idealized and simplified model of rectangle structure.** The top view of DAS model of Si(111)-7 × 7-reconstructed surface **(a)** and the idealized and simplified model of rectangle structure with periodicity **(b)**. The red and blue line was the length and width of rectangle. In order to show clearly the relationship between rectangular Fe cluster and Si(111)-7 × 7-reconstructed surface, the C_2_H_5_OH layer was not shown in (b).

In general, the periodical arrangement of Si atoms on Si(111)-7 × 7-reconstructed surface could result in the periodical surface potential field [20-22]. Then, the periodical surface potential field could restrain the growth of Fe cluster with certain periodicity. Based on the dimer-ad-atom-stacking (DAS) model of Si(111)-7 × 7-reconstructed surface [23], the side length of unit cell was 2.668 nm, just as shown in Figure 5a and the rhombus A in Figure 5b. According to the periodicity of rhombus unit cell in DAS model, the smallest rectangle structure with periodicity could be designed as the rectangle B-E shown in Figure 5b. Through the simple calculation, the width and length of the designed rectangle was 4.66 and 5.376 nm, respectively, which was corresponded well with the values of Fe clusters in Figures 2d and 4. The center of symmetry for rectangle B-D in Figure 5b was totally the same. But, for the rectangle E, the center of symmetry was different. Besides, it should be noticed that the length of the designed rectangle was 15% more than the width. Based on the hypothesis of Fe cluster with single-domain structure, the amount of magnetic lines through the common length side of two adjacent rectangular Fe clusters (B-D) were more than the magnetic lines through the common width side (B-C). So, instead of B-C direction, the rectangular Fe clusters were linked along B-D direction, preferentially. By controlling the interval between the straightly linked chains, the Fe clusters with critical size of 5 nm prepared by our technique could be one of ideal candidates for high-density magnetic recording medium.

## Conclusions

In summary, we attained to control the preparation of 5-nm Fe clusters on Si(111)-7 × 7-C_2_H_5_OH surface. The Fe cluster is stabilized by the interaction with Si ad-atoms with a dangling bond remained on the Si(111)-7 × 7-C_2_H_5_OH surface. The periodical arrangement of Si atoms on Si(111)-7 × 7-reconstructed surface and the periodical surface potential field restrained the growth of Fe clusters with certain periodicity. The XPS results showed that the Fe clusters were stable in the thin-air condition (4.5 × 10^-2^ Langmuir) at room temperature. When the deposition of Fe atoms was increased, about-5-nm Fe clusters were formed and underwent one-dimensional self-assembly crossing the step onto the upper or lower terrace. The driving force making one-dimensional linked straight chain structure might be the magnetic force of Fe clusters. If so, the Fe cluster takes single magnetic domain with about 5 nm of critical size, and we could expect to lower the single magnetic domain to ca. 5 nm without a change to the super paramagnetic property. Based on our results, the Fe cluster is hopefully to synthesize the strong magnetic FeN_*x*_ and FeO_*x*_ particles with 5 nm of critical size in the future. Finally, from the point of applying Fe clusters as the high-density magnetic recording medium, it is interesting to prepare the Fe clusters with a critical size lower than 10 nm. The present work reveals a simple way to realize it as well as the physicochemical mechanism behind it.

## Abbreviations

STM: scanning tunneling microscopy; XPS: X-ray photoelectron spectroscopy; ML: mono layer; FWHM: full width at half-maximum; DAS: dimer-ad-atom-stacking.

## Competing interests

The authors declare that they have no competing interests.

## Authors' contributions

DJ conceived of the idea. KT designed the STM experiment and gave suggestions on the preparation of the sample. WD carried out the STM experiment, analyzed the data, and drafted the manuscript. YG carried out the XPS measurement. DJ, KT, and FK participated in the analysis of results. All authors read and approved the final manuscript.
